# Evaluation of the Caries-Arresting Potential of Silver Diamine Fluoride Gel With Potassium Iodide Versus Sodium Fluoride Varnish: An Observational Study

**DOI:** 10.7759/cureus.64970

**Published:** 2024-07-20

**Authors:** Ayesha Fathima, Lavanya Govindaraju

**Affiliations:** 1 Pediatric Dentistry, Saveetha Dental College and Hospitals, Saveetha Institute of Medical and Technical Sciences, Saveetha University, Chennai, IND

**Keywords:** preventive dentisry, silver diamine fluoride, non-invasive management, fluoride varnish, caries arresting agent

## Abstract

Introduction

Silver diamine fluoride (SDF) with potassium iodide (KI) has emerged as a promising, aesthetic, non-invasive management by limiting the staining caused by SDF. However, no studies are comparing the caries-arresting potential of SDF gel with KI. The study aims to observe and evaluate the caries-arresting potential of SDF gel with KI compared to sodium fluoride (NaF) varnish.

Materials and methods

The present observational study was conducted with a split-mouth design. A total of 33 participants, in the age group of two to six years, with occlusal caries of the International Caries Detection and Assessment System (ICDAS) I and II involving both right and left upper or lower primary molar teeth, were included. SDF gel with KI and fluoride varnish (FV) was applied to the contralateral teeth on the same arch. Participants were recalled at 3, 6, 9, and 12‐month intervals to monitor the progression or arrest of carious lesions.

Result

At the end of 12 months, 66.7% and 70.5% of the teeth showed arrest of caries with SDF gel with KI and FV, respectively. Intra-group analysis using the Friedman test shows significant differences in both groups across the timeline (p=0.001). Intergroup analysis using the Whitney U test reveals no significant difference in caries-arresting potential between the groups at various timelines (p=0.231).

Conclusion

There was no significant difference in arresting enamel caries of primary teeth between the use of 5% NaF and 38% SDF with KI. However, there was a significant difference within the group between the 3rd and 12th-month follow-up. Hence, the biannual application is recommended in both FV and SDF with KI.

## Introduction

Dental caries, a common chronic disease in children, has increased globally, especially in developing countries, with over 570 million affected children worldwide [1,2]. It is a preventable, biofilm-mediated disease and is strongly linked to behaviour, diet, and hygiene habits. Control and prevention are possible by reducing the fermentable carbohydrates in the diet and disrupting dental biofilm systematically [3,4]. Early childhood caries (ECC) in children under 71 months is influenced by socioeconomic, dietary, oral health, and biological factors, leading to decayed, missing, and filled tooth surfaces [5,6].

Fluoride varnish (FV) and silver diamine fluoride (SDF) are used in paediatric dentistry as non-invasive caries treatment techniques in non-cavitated carious lesions to treat children as young as one to four years old [7]. FV is a topical fluoride agent commonly used for caries prevention in children. It has remineralisation and antimicrobial properties, making it the gold-standard agent for preventing dental caries in children [8]. SDF is another non-invasive agent that has received renewed interest in the current decade. It is an efficient, affordable, equitable, and effective cariostatic agent [9]. This non-restorative approach can halt the progression of carious lesions and can be an alternative to control the burden of dental caries in children worldwide [10]. This is an interesting treatment approach for deciduous teeth that is recommended by the American Academy of Pediatric Dentistry in 2017 [11]. SDF was approved by the Food and Drug Administration of the United States in 2014. Since then, there has been growing interest in its “off‐label” use for arresting carious lesions in children. There is a multitude of benefits with SDF in caries management. It is cost-effective, non‐aerosol generating, and does not require any dental equipment [12]. 

However, the critical disadvantage of the application of SDF is the discolouration of teeth. Aly and Yousry reported that enamel and dentin, including carious tissues, are decolourized into dark brown or black after SDF application [13]. The greater the extent of demineralization, the more the silver ions are absorbed, thereby increasing discolouration and the distinction between affected and sound tissues. The stain remains over time and can only be removed through physical methods [14]. Research has explored caries-arresting potential and comparative studies with SDF solutions [9-11]. However, in recent years, gel-based SDF in augmentation with potassium iodide (KI), like Kedo SDF gel, was introduced to dentistry to achieve caries prevention. Therefore, the primary aim of the present study was to observe and evaluate the effect of 38% SDF gel and KI versus 5% sodium fluoride (NaF) varnish on arresting the progression of incipient carious lesions in children.

## Materials and methods

Study setting and sample size calculation

The present observational study was conducted on children brought to the Pediatric and Preventive Dentistry Outpatient Department at a private dental institution in Chennai, India. It was an observational cohort study where the natural progression of dental caries and the use of treatments already in place were monitored. The study was approved by the Institutional Review Board and Ethical Committee, and adhered to the ethical standards laid down by the Helsinki Declaration. The study was registered with the Institutional Human Ethical Committee at Saveetha University (IHEC/SDC/PEDO-2104/23/105).

The sample size was calculated based on the results of the study done by Mani Prakash et al. [3], using the G*Power software, version 3.1.9.7 (Heinrich-Heine-Universität Düsseldorf, Düsseldorf, Germany). At a level of significance set at 5%, the power of the study at 95%, and for an expected critical Z-value of 3.193, it was calculated that 48 teeth, i.e., 24 teeth per group, were required to perform the study. Finally, a sample size of 33 teeth was taken in each group, assuming a 40% loss to follow‐up.

Inclusion and exclusion criteria

Cooperative children with Frankl III (positive) and IV (definitely positive) behaviour, in the age group of two to six years, with occlusal caries of the International Caries Detection and Assessment System (ICDAS) I and II involving both right and left upper or lower primary molar teeth, without any pulpal involvement, and willing to participate in the study, were included. Children who are medically compromised, allergic to silver, or participating in any other studies were excluded.

Data collection and treatment observation

Participants underwent a baseline examination to determine their eligibility for inclusion in the study. A total of 33 children, with 66 carious primary molars, were included in the study. These children were already undergoing treatment with either SDF followed by KI or NaF varnish, based on clinical judgment and parental preference.

In cases where SDF followed by KI was applied, Vaseline was applied on all other surfaces of the tooth and gingiva, except the occlusal surface, to avoid SDF staining. The selected teeth were cleaned and dried for five seconds, isolation was done using cotton rolls, and 38% SDF gel was applied over the cavitated lesion directly from the syringe for 60 seconds, followed by KI with a micro applicator tip. The precipitate was then cleaned using a moistened cotton pellet.

**Figure 1:**
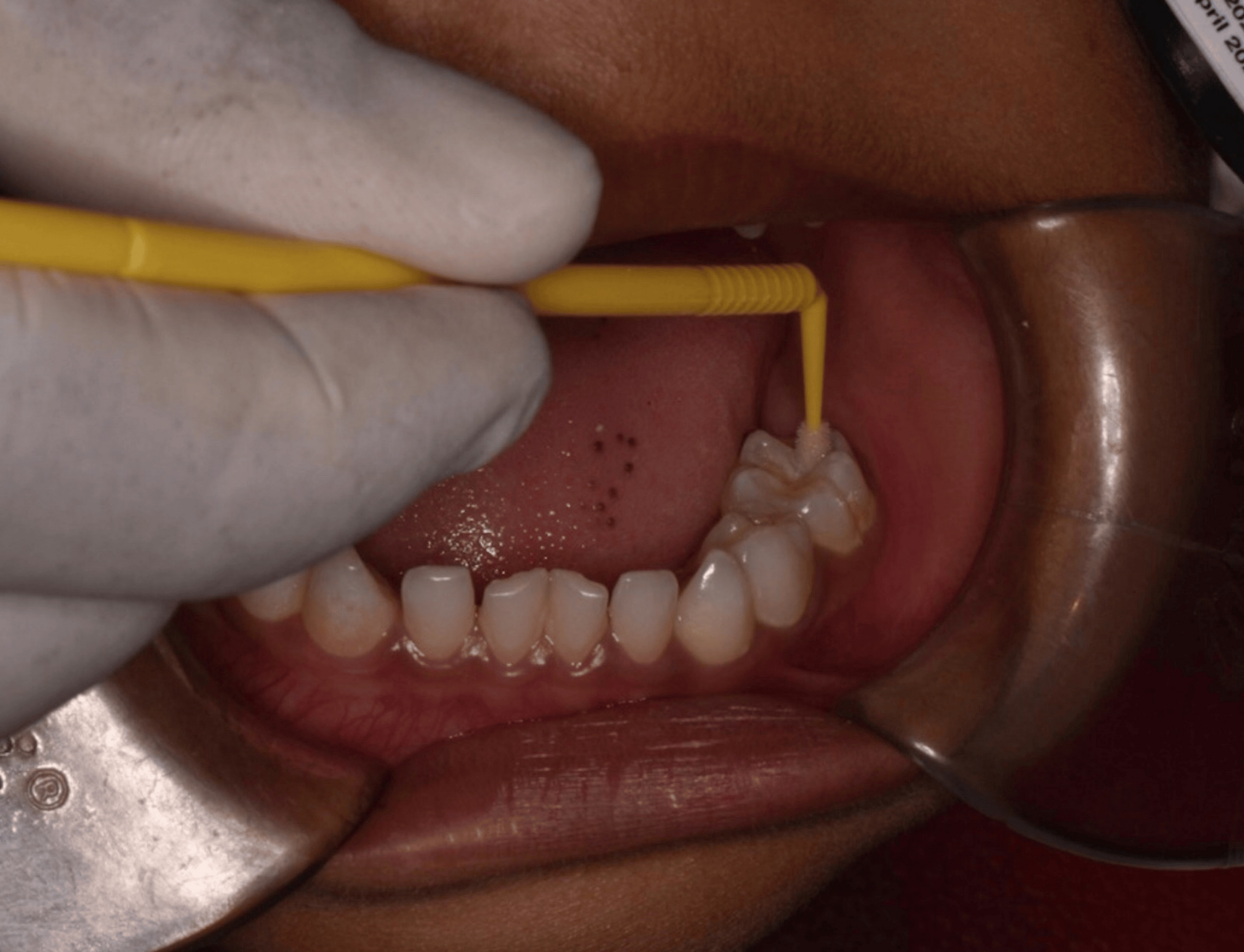
Application of SDF gel using applicator tip on the occlusal surface of the primary second molar. SDF: Silver diamine fluoride

**Figure 2:**
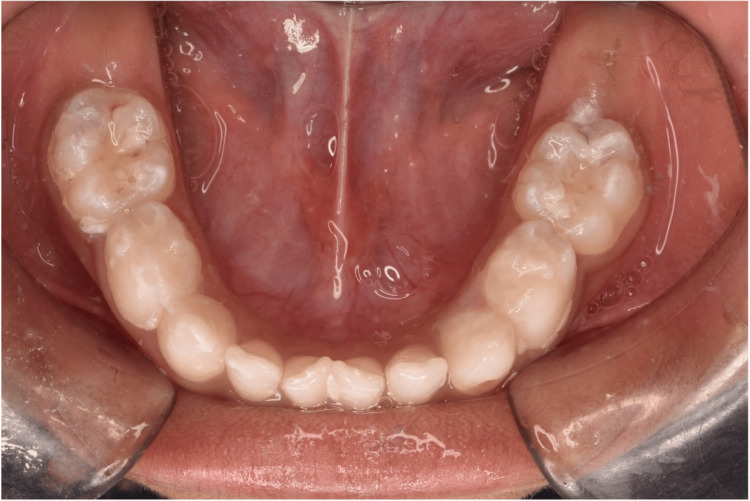
Post application of SDF gel with KI on 75 and fluoride varnish on 85. A white precipitate formed over the occlusal surface of 75 on the application of KI to discolour the SDF stain. SDF: Silver diamine fluoride; KI: Potassium iodide

In cases where NaF varnish was applied, the selected teeth were cleaned and dried for five seconds. Isolation was done using cotton rolls, and topical NaF varnish containing 5% NaF was applied using a micro applicator tip and allowed to dry for one minute. Patients were advised not to drink or eat for 30 minutes, and oral hygiene instructions were given (Figures 1-2).

Follow-up and evaluation

The children were observed periodically at 3, 6, 9, and 12-month intervals. Two blinded evaluators evaluated the progression or arrest of caries. During these follow-up visits, if the cavity wall or floor was easily penetrated by the Community Periodontal Index of Treatment Needs (CPITN) probe using light force, it was diagnosed with active caries. Cavities with hard surfaces were classified as arrested caries. Two clinicians were trained to evaluate caries, and inter-examiner variability was checked to avoid bias. The clinicians evaluated the caries-arresting potential and any incidence of new caries on the other surfaces of the primary molars.

Statistical analysis

The data was analysed using IBM SPSS Statistics for Windows, Version 23 (Released 2015; IBM Corp., Armonk, NY, USA). Caries increment was described as frequency and percentages. Shapiro-Wilk test was done to assess the normality of the data. Mann-Whitney U test was done to assess the differences in the caries presence between the two groups with specific timelines. Within the group, the caries presence and change were assessed across the timeline by the Friedman test, with a significance level of less than 0.05.

## Results

A total of 33 children, with 66 teeth, received both SDF gel with KI and FV in their contralateral primary molars as a non-invasive treatment measure to arrest dental caries. The mean age of the participants was 3.24 ± 0.9 years. In the third month, the arrested caries were 100% intact in both groups, whereas in the sixth month, they dropped to 93.9% in the SDF gel and 97% in the NaF varnish. Further decline was observed with 72.7% in the SDF gel and 73.5% in the NaF varnish group. At the end of 12 months, about 66.7% of the teeth showed arrest of caries with SDF gel and about 70.5% of the teeth showed arrest of caries with NaF varnish. Intra-group analysis showed that both SDF gel and NaF varnish are significantly effective in arresting caries across various timelines (Table 1).

**Table 1:** Intragroup analysis on the effectiveness of caries-arresting potential between the groups across various time intervals using the Friedman test (p<0.05 indicates significance) SDF: Silver diamine fluoride

Groups	3 months	6 months	9 months	12 months	p-value
SDF	100	93.9	72.7	66.7	<0.001
Varnish	100	97	73.5	70.5	<0.001

Intergroup analysis between both groups at various timelines, comparing caries-arresting potential and caries increment at different time intervals, was conducted using the Mann-Whitney U test. The results show no significant difference between the groups at baseline, 3, 6, 9, and 12 months (Table 2).

**Table 2:** Intergroup analysis on the comparison of caries-arresting potential and caries increment between the groups at various time intervals (Mann-Whitney U test). SDF: Silver diamine fluoride

Follow-up timelines	Groups	Caries intact n(%)	Caries increment n(%)	Mann-Whitney U-value	p-value
3 months	SDF	33 (100)	0 (0)	544.5	1.000
	Varnish	33 (100)	0 (0)		
6 months	SDF	31 (93.9)	2 (6.1)	528	0.956
	Varnish	32 (96.9)	1 (2.9)		
9 months	SDF	24 (72.7)	9 (27.3)	526.5	0.651
	Varnish	25 (73.5)	8 (26.5)		
12 months	SDF	22 (66.7)	11 (33.3)	510.56	0.231
	Varnish	24 (70.5)	9 (29.4)		

## Discussion

The efficacy of SDF in caries-arresting potential lies in its multifaceted mechanism of action. Silver ions exhibit potent antimicrobial properties, targeting and inhibiting the growth of cariogenic bacteria within dental biofilms [15]. Additionally, fluoride ions promote remineralization of the demineralized tooth structure, strengthening the enamel and dentin, and thereby reducing the susceptibility to caries [16]. The choice of study design and the implementation of this clinical trial was conducted by enforcing non-invasive caries-arresting treatment measures, which could impact carious children at a very young age and also for public health purposes, which do not require much armamentarium and dental setup [17]. The present study is the first split-mouth observational study to compare the effectiveness of 38% SDF gel with KI to 5% NaF varnish on the occlusal surfaces of the primary molars. In this clinical trial, FV was used as a control, which is widely recognized as the standard of care for preventing caries [18,19].

With regards to the caries-arresting potential, intragroup analysis within the group across various timelines shows both SDF in gel form and FV are equally effective in arresting the caries till three months following the application, after which both the groups show a gradual decline, with FV having slightly higher caries-arresting potential than SDF gel by 12 months. However, in the intergroup analysis, there was no significant difference noticed between both groups. The results of the present study were in accordance with the systematic review that concluded that FV had a modest and uncertain effect in preventing caries development in children [14,20]. Another systematic review and meta-analysis comparing the non-restorative treatments for caries stated that 5% NaF varnish was the most effective for arresting or reversing non-cavitated facial/lingual carious lesions and that 38% SDF solution was the most effective for arresting carious lesions on any coronal surface only when applied biannually [21]. Consistent with the present study, a randomized controlled trial by Gao et al. stated that there were no differences in the caries preventive effect between SDF and FV on bilateral molar teeth [22]. Earlier clinical studies have reported that the caries-arresting rate increases over time when the interventions were repeated annually or semi-annually [23,24].

The limitations of the present study include the intervals between the dental examinations, which were every three months. Therefore, the exact time when the carious lesions were arrested cannot be provided. Secondly, the study design used the Last Observation Carried Forward (LOCF) imputation method, because of which the caries-arresting effectiveness reported in the present study might be lower than the true number. Also, cooperation from very young children, as young as two years, for isolation cannot be expected. In the present study, SDF gel with KI and FV was applied on the contra-lateral molars in the same visit, and the synergistic effect of both might have also been involved in arresting the progression of the caries.

The future scope of this study includes conducting long-term follow-ups with more participants to validate the findings across diverse populations, and advanced diagnostic tools and imaging techniques could be employed to monitor carious lesions more precisely. Further research should investigate the effectiveness of other caries-arrest agents and combinations, as well as the impact of dietary habits and oral hygiene practices on treatment outcomes. Evaluating the long-term sustainability and benefits of caries-arrest treatments, and developing educational programs for patients and caregivers, will also contribute to improving the understanding and effectiveness of caries management in young children.

## Conclusions

Based on the above results, there was no significant difference in arresting enamel caries of primary teeth between the use of 5% NaF and 38% SDF gel with KI. However, there is a significant difference within the group between the 3rd-month and 12th-month follow-up. Hence, biannual application is recommended in both FV and SDF gel with KI.
